# Contributions to the Knowledge of Nemognathinae (Coleoptera: Meloidae) from China†

**DOI:** 10.3390/insects15050338

**Published:** 2024-05-08

**Authors:** Shaopeng Wang, Yinuo Sun, Liang Lü, Zhao Pan

**Affiliations:** 1 Key Laboratory of Zoological Systematics and Application of Hebei Province, School of Life Sciences, Institute of Life Science and Green Development, Hebei University, Baoding 071002, China; wangshaopeng99@yeah.net; 2 Key Laboratory of Animal Physiology, Biochemistry and Molecular Biology of Hebei Province, College of Life Sciences, Hebei Normal University, Shijiazhuang 050024, China; sunyinuo6@163.com (Y.S.); lianges.luex@gmail.com (L.L.)

**Keywords:** blister beetles, new genus, new species, new faunistic records, molecular phylogenetics, China, taxonomy

## Abstract

**Simple Summary:**

Nemognathinae is notable for its high diversity in Meloidae, yet our knowledge about its species in China remains restricted. Here, we describe a new genus and species and report three other species as new additions to the Chinese fauna. Based on our investigation of the types, we transfer the genus *Oreomeloe* from Meloini to Nemognathini. Finally, we present molecular phylogenies built on multi-locus data, which corroborate the systematic placement of *Sinostenoria* and *Longizonitis*. Our findings and treatment contribute to the understanding of Chinese Nemognathinae and resolve a pending issue in the taxonomy of Nemognathinae.

**Abstract:**

Despite being the most widespread blister beetle subfamily, Nemognathinae is unfairly understudied in China. In this study, a new genus and species, *Sinostenoria yangi* Pan, from northern China is described and illustrated. The antennae, elytra, hind wings, and claws of the new genus form a truly unique set of characteristics never observed in other genera of Nemognathini Laporte de Castelnau, 1840. Three species from China are newly recorded and illustrated: *Megatrachelus sibiricus* (Tauscher, 1812), *Zonitomorpha dollei* (Fairmaire 1889), and *Stenodera djakonovi* Aksentjev, 1978. The genus *Oreomeloe* Tan, 1981, is transferred from the tribe Meloini Gyllenhal, 1910, to Nemognathini based on an examination of the types. Aiming to test the morphology-based placement of the new genus, we conducted molecular phylogenetic analyses using two mitochondrial (*COI*, *16S*) and three nuclear markers (*28S*, *CAD*, *ITS2*). The results confirm our tribal assignment of the new genus and support a clade that consists of *Sinostenoria* **gen. n.**, *Longizonitis* Pan and Bologna, 2018, *Stenoria* cf. *grandiceps*, and *Ctenopus* cf. *persicus*.

## 1. Introduction

The blister beetle subfamily Nemognathinae Laporte de Castelnau, 1840, consists of 5 tribes, 34 genera, and almost 600 species, distributed throughout the world except for New Zealand, the eastern Polynesian Islands, and Antarctica [1,2]. Molecular studies using selected genes [2,3] and genomics [4] have recently provided evidence for the monophyly of this subfamily. No comprehensive taxonomic revision or phylogenetic studies had been published on this subfamily, until Riccieri et al. [2] described the evolutionary history of Nemognathinae based on molecular data. Nevertheless, the taxonomic issues uncovered by their phylogenetic results remained unresolved [1,2,5,6,7].

Despite having a high diversity of genera and species, there is limited knowledge on Nemognathinae from China. Currently, 35 species, belonging to 14 genera of 3 tribes, have been documented from China (see Appendix A) [8,9]. During the taxonomic investigation of the Chinese Meloidae, we present here three findings concerning Nemognathinae: (a) one new genus (*Sinostenoria* Pan, **gen. nov.**), one new species (*Sinostenoria yangi* Pan, **sp. nov.**); (b) three species new for China; and (c) the misplacement of *Oreomeloe* Tan, 1981. We then transferred the genus from Meloini Gyllenhal, 1810 (subfamily Meloinae Gyllenhal, 1810), to Nemognathini Laporte de Castelnau, 1840. Additionally, molecular phylogenetic analyses were implemented using both mitochondrial and nuclear genes to determine the placement of *Sinostenoria* **gen. nov**. and *Longizonitis* Pan and Bologna, 2018, which were not included in the previous analyses conducted by Riccieri et al. [2].

## 2. Materials and Methods

### 2.1. Morphological Study

A complex of 66 adult nemognathine specimens were examined for this study: the holotype and 16 paratypes of *Sinostenoria yangi* **gen. and sp. nov.**; 6 exx. (including 1 syntype) of *Zonitomorpha dollei*; 38 exx. of *Megatrachelus sibiricus*; 5 exx. of *Stenodera djakonovi*; and the holotype and 2 paratypes of *Oreomeloe spinulus*. The following abbreviations were used to represent the studied collections (acronyms of collections in alphabetical order): CAU = Entomological Museum of China Agricultural University, Beijing, China; NACRC = National Animal Collection Resource Center, Beijing, China; MHBU = Museum of Hebei University, Baoding, China (MHBUa = the material preserved in alcohol 95%); MNHN = Muséum National d’Histoire Naturelle, Paris, France; NKU = Entomological Museum of Nankai University, Tianjin, China; ZIN = Russian Academy of Sciences, Zoological Institute, St. Petersburg, Russia.

Most of the photos were captured with a Canon EOS 5D Mark III camera (Canon Inc., Tokyo, Japan) mounted on a Laowa FF 100 mm F2.8 CA-Dreamer Macro 2× or Laowa FF 25 mm F2.8 Ultra Macro 2.5–5× (Anhui Changgeng Optics Technology Co., Ltd., Hefei, China). The photographs of the holotype of *Oreomeloe spinulus* were taken by Chunyan Jiang (NACRC), but those of its antenna, tibial spurs, and pretarsal claws were taken by one of the authors (Z. P.) using the embedded camera of a Xiaomi 12 mobile phone.

The anatomical terminology used in the descriptions are the same as those in previous studies on Nemognathinae [1,6]. The body length was measured from the labrum through the apex of abdomen. The body width was measured at the base of elytra. Label data of the type materials were copied verbatim. Line breaks on labels are denoted by a double slash (//); metadata and notes (not printed on the labels themselves) are indicated in square brackets ([]). Scientific names are uniformly presented in italics.

### 2.2. Molecular Taxon Sampling, DNA Extraction, Amplification, Sequencing, and Sequence Editing and Alignment

For the purpose of updating the phylogeny of Nemognathinae and resolving the relationships of *Sinostenoria* and *Longizonitis*, two individuals (one of *Sinostenoria yangi* and one of *Longizonitis semirubra*; the details are provided in Table S1) were selected and sequenced. In addition, sequences of 91 individuals of 54 species (including the outgroup *Prolytta coriacea*), previously published by Riccieri et al. [2,3], were selected and downloaded from GenBank (see Table S2 in Riccieri et al. [2]).

The genomic DNA of *Sinostenoria yangi* and *Longizonitis semirubra* was extracted from muscles of the thorax and legs from one side using an Insect gDNA Isolation Kit (BIOMIGA^®^, Hangzhou, China). Complete genomes were sequenced by an illumina NextSeq CN500 (BerryGenomics, Beijing, China) to obtain raw sequence data. The mitochondrial genome was assembled by using MitoZ v3.4 or NOVOPlasty v4.3.1 and then was annotated in MitoZ v3.4 [10,11]. The cytochrome c oxidase subunit I (*COI*) and mitochondrial 16S ribosomal RNA gene (*16S rRNA*) were extracted from the whole sequence of mitochondrial genomes. Three nuclear gene fragments were obtained by Sanger sequencing with specific primers: 28S ribosomal RNA (*28S rRNA*; primer pairs: 28S01/28SR01 [12]); carbamoylphosphate synthetase domain of the rudimentary gene (*CAD*; primer pairs: CD439F/CD688R; CD439F/CD668R [13]); and internal transcriber spacer 2 (*ITS2*; primer pairs: ITS2-3d/ITS2-4r [14]). Polymerase chain reaction (PCR) amplifications were performed with the settings used in Riccieri et al. [2]. The PCR products were examined using 1.0% agarose gel electrophoresis and purified and sequenced by General Biol (Chuzhou, China). The nuclear gene sequences were edited in DNASTAR SeqMan Pro v.7.1.0 (DNASTAR, Inc., Madison, WI, USA).

All Sequences were checked using BLASTN in NCBI [15,16,17,18,19,20,21,22], aligned using MAFFT v7.0 [23], and trimmed by trimAl [24].

### 2.3. Molecular Phylogenetic Analyses

Phylogenetic trees were constructed using the Maximum Likelihood (ML) method and Bayesian Inference (BI) with the concatenated alignments of the five gene fragments (*COI*, *16S*, *28S*, *CAD*, *ITS2*) of all available specimens.

The ML analysis was performed using IQ-TREE v1.6.8 [25]. Data were partitioned depending on genes. The best partition scheme and the best substitution model of each partition were selected by ModelFinder [26] according to the corrected Akaike information criterion (AICc), considering the edge-linked method. An Ultrafast Bootstrap [27] of ten thousand repeats was carried out for the nodal supports. The minimum correlation coefficient was 0.90. Tree analysis was run 10 times. BI analysis was conducted using MrBayes v3.2.6 [28]. The best partitioning scheme was the same as IQ-TREE. An edge-linked model was considered. MCMC sampling was run by 20 million generations and saved every 1000 generations. The convergence of the chains was assessed using Tracer v1.7 [29] and the first 25% of samples were discarded before summarizing the MCMC samples. The final trees were visualized using FigTree v1.4.4 [30].

## 3. Results

The complete genomic raw datasets of *Sinostenoria yangi* and *Longizonitis semirubra* are both 14 Gb, respectively. The mitochondrial genomes obtained consist of 15,607 bp for *L. semirubra* and 15,606 bp for *S. yangi*. The sequences of *S. yangi* and *L. semirubra* have distinct lengths in terms of bases pairs (bp): *COI* (638 bp both species), *16S* (441 bp both), *28S* (906 bp *S. yangi* and 897 bp *L. semirubra*), *CAD* (896 bp *S. yangi* and 862 bp *L. semirubra*), and *ITS2* (520 bp *S. yangi* but failed to amplify in *L. semirubra*).

### 3.1. Molecular Phylogenetics

**Figure 1:**
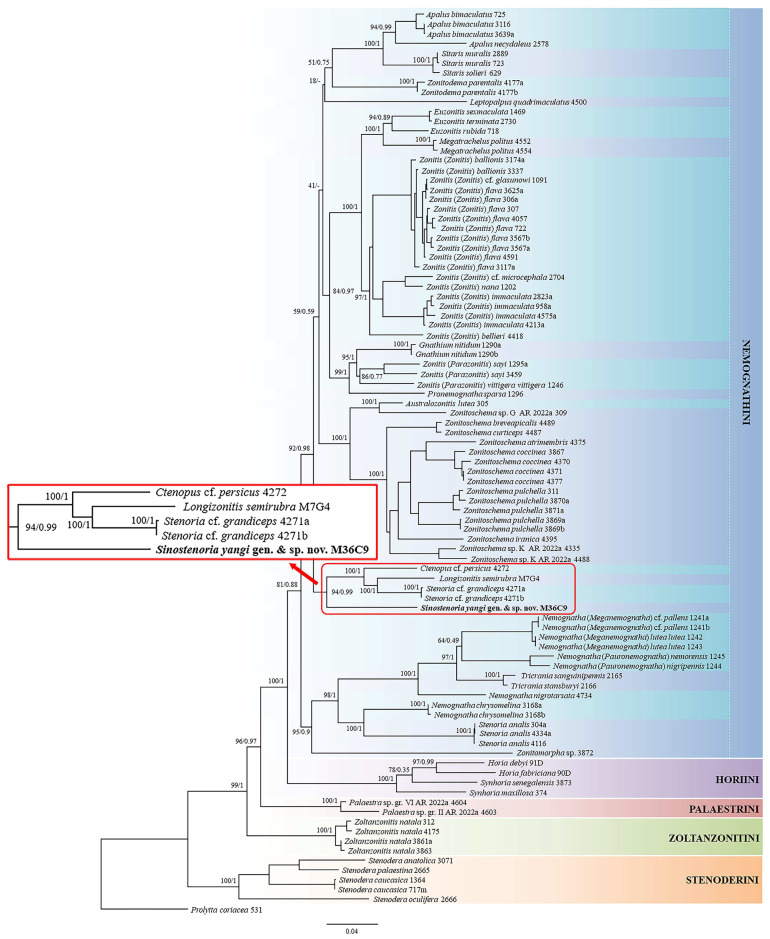
Multi-locus (*16S*, *COI*, *CAD*, *ITS2*, and *28S*) phylogenetic tree of Nemognathinae. Topology corresponds to the Maximum Likelihood (ML) tree. Tribes are highlighted with different colors. Support for each node is represented by Ultrafast Bootstrap values (uBV, %) and Bayesian posterior probability (bpp). Dashes (-) indicate non-supported values.

Both the BI tree and ML tree exhibit identical topology with strong node supports (Figure 1). Both *Sinostenoria* **gen. nov.** and *Longizonitis* unequivocally belong to the tribe Nemognathini. Regarding the relationships among genera, these two genera, along with *Ctenopus* cf. *persicus* Semenov, 1893 and *Stenoria* cf. *grandiceps* (Semenov, 1893), form a distinct clade within Nemognathini [Ultrafast Bootstrap value (uBV, %) = 94; Bayesian posterior probability (bpp) = 0.99]. As reported by Riccieri et al. [2], the genus *Stenoria*, in the present definition, is polyphyletic, and *S.* cfr. *grandiceps* may actually belong to a different genus. However, due to the limited information available on the taxonomy and systematics of this genus, it is not currently possible to resolve this issue. Within this clade, *Sinostenoria* is fundamentally different from the other species, but *Longizonitis* is closely related to the two samples of *Stenoria* cf. *grandiceps* (uBV = 100; bpp = 1).

### 3.2. Description of New Taxa

**Genus *Sinostenoria* Pan, gen. nov.**

**Type species.** *Sinostenoria yangi* Pan, **sp. nov.**, by monotypy and present designation.

**Diagnosis.** The new genus belongs to the lineage ‘Sitarini’ [2] due to the following characteristics: maxillary palpi normal length; elytra reduced in apical width and in length as well; ventral blade of claws narrow, its greatest width less than half basal width of dorsal blade. However, the taxonomy of the lineage ‘Sitarini’ is totally unresolved also at the genus level, and consequently, the relationships are not delineable. The new genus could be distinguished by the combination of the following characteristics: male with 11 antennomeres but female only with 10 antennomeres; elytra abbreviated, not immediately dehiscent at base; hind wings reduced and unfolded, completely covered by elytra; dorsal blade of claws smooth along ventral margin, and ventral blade of claws setiform. The details are given in the Discussion below.

**Figure 2:**
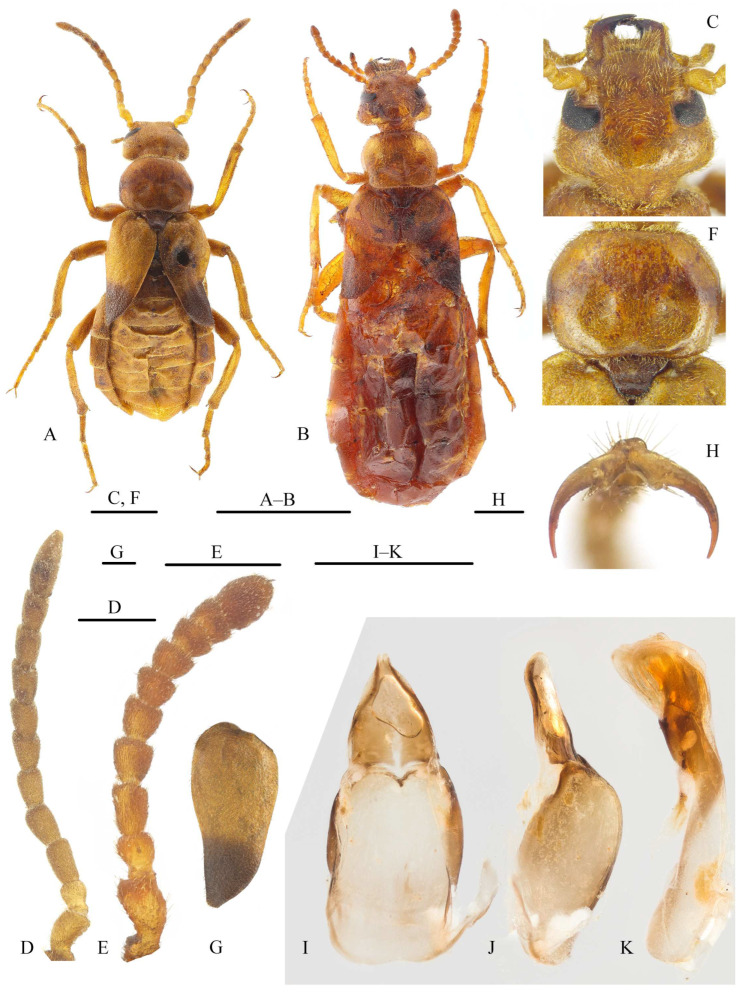
*Sinostenoria yangi* Pan, **gen. and sp. n.** (**A**,**B**) Habitus, dorsal view; (**C**) head, dorsal view; (**D**,**E**) antennae, dorsal view; (**F**) pronotum, dorsal view; (**G**) left elytron, dorsal view; (**H**) pretarsal claws of fore leg; (**I**,**J**) tegmen: (**I**) ventral view, (**J**) lateral view; (**K**) aedeagus, lateral view. (**A**,**C**,**D**,**F**–**K**) male, holotype; (**B**,**E**) female, paratype (from Ningxia, Yanchi). Scale bars: 5 mm (**A**,**B**); 1 mm (**C**–**G**,**I**–**K**); 0.2 mm (**H**).

**Description.** Head (Figure 2C) subtriangular, with maximum width at level of temples; temples prominent; occiput protruded. Eyes relatively small; minimum distance between inner margins of eyes distinctly wider than width of each eye in dorsal view. Fronto-clypeal suture invisible. Labrum transverse; mandibles robust, extending beyond anterior margin of labrum; maxillary galeae penicillate and short, palpi four-segmented, palpomeres robust, IV not widened at apex. Antennae sexually dimorphic; male antennae (Figure 2D) relatively longer, with 11 antennomeres; female antennae (Figure 2E) distinctly shorter than male, with 10 antennomeres, due to fusion of last two not being visible.

Pronotum (Figure 2F) distinctly wider than long, lateral sides curved. Scutellum well visible. Elytra (Figure 2G) short, about as long as half of abdomen in male and less in female, not completely covering abdomen, dehiscent a few after scutellum, quite lyriform and sub-arcuate on lateral margins, especially in male. Hind wings reduced, shorter than elytra, unfolded, completely covered by elytra. Legs not modified; metatibial spurs similar in shape; dorsal blade of pretarsal claws smooth along ventral margin, ventral blade narrow and short, its greatest width distinctly less than half basal width of dorsal blade and its length distinctly shorter than half length of dorsal blade (Figure 2H).

Posterior margin of ventrite V straight; ventrite Ⅵ longitudinally divided into two lobes in male and only slightly curved inwards on posterior margin of female. Male gonoforceps (Figure 2I,J) almost completely fused, slightly separate at apex; gonocoxal plate distinctly longer and wider than gonoforceps. Aedeagus subcylindrical, without dorsal hooks; endophallus without hook.

**Etymology.** From the Latin root “*sino*-” for “China” and *Stenoria*. This generic name is feminine.

**Distribution.** N China.

***Sinostenoria yangi* Pan, sp. nov.** (Figure 2)

**Description.** Body (Figure 2A,B) shiny brownish yellow, except mandibular apical half and elytral apical third, which dark brown to black, and pretarsal claws dark. Body covered by short yellow setae. Body length: 11.5–15.9 mm; width: 3.8–4.9 mm.

Head (Figure 2C) wider than long (ca. 0.77× as long as wide), with maximum width at level of temples, with dense, small punctures; diameter of punctures similar to intermediate spacing among punctures. Frons flat, slightly convex on inner side of antennal socket. Temples prominent; occiput protruded. Eyes relatively small; minimum distance between inner margins of eyes approximately twice width of each eye in dorsal view. Clypeus short, subtrapezoidal. Labrum inverted trapezoidal, anterior margin distinctly emarginate in middle; mandibles approximately as long as half of head in lateral view; galeae shorter than maxillary palpi, palpomere II longest and distinctly widened apically, III–IV subequal in length and width. Male antennae (Figure 2D) posteriorly reaching middle of elytra: antennomere I robust; II sub-moniliform, shortest; III–XI longer than wide; III–VI similar in shape, slightly widened apically; III approximately as long as I and longer than others, except XI; IV–VI subequal in length; VII–X subcylindrical; XI longest, subfusiform, widest near base. Female antennae (Figure 2E) shorter than in male, only posteriorly reaching base of pronotum: I and II similar to male; III longest, except I and X, longer than wide, and slightly widened at apex; IV–IX distinctly widened at apex; IV slightly longer than wide and approximately as long as II; V shorter than IV and approximately as long as wide; VI and VII subequal in length and shorter than V; VIII and IX subequal in length and shorter than VII; X shorter than I and slightly longer than III, suboval.

Pronotum (Figure 2F) approximately 0.75× as long as wide, lateral sides curved, widest in front of middle, converging progressively to apex and base, posterior margin emarginated in middle; dorsal punctures smaller and sparser than those on head, almost invisible; disc with one rounded shallow depression on each side. Prosternum with a transverse carina in front of procoxal cavity. Scutellum subtriangular, posterior margin rounded. Elytra (Figure 2G) short, posteriorly reaching abdominal segment III. Legs slender; tibiae almost straight, pro- and mesotibial spurs slender and pointed, metatibial spurs wider, obtuse at apex; tarsomere I longest, last one second longest; ventral blade of pretarsal spine-shaped (Figure 2H). Tarsomeres (except last one) and apical half of protibiae with pads.

Eight abdominal tergites visible. Male gonoforceps (Figure 2I,J) short, subtriangular in ventral view, approximately as half-length of gonocoxal plate; apex of lobes ventrally curved in lateral view. Aedeagus as in Figure 2K.

**Diagnosis.** This species, the only known species of *Sinostenoria*, can be recognized by the generic diagnosis given above.

**Type locality.** Ming Dynasty Tombs, Beijing City, China.

**Type specimens.** Holotype: male, with the following labels: “北京农业大学昆虫学系 [Beijing Agricultural University, Department of Entomology]//北京十三陵 [China, Beijing, Ming Dynasty Tombs]//杨集昆 [Jikun Yang leg.], 1956.VII.24”, “HOLOTYPE//*Sinostenoria yangi* gen. and sp. nov.//Det. Pan Z.” (CAU).

Paratypes: one male, labeled “北京农业大学昆虫学系 [Beijing Agricultural University, Department of Entomology]//北京十三陵 [China, Beijing, Ming Dynasty Tombs]//杨集昆 [Jikun Yang leg.], 1956.VII.24” (CAU); one male, labeled “1956-VII-23//昌平十三陵 [China, Beijing, Changping, Ming Dynasty Tombs]//王槐青 [Huaiqing Wang leg.]” (CAU); one male, labeled “1956-VII-24//昌平十三陵 [China, Beijing, Changping, Ming Dynasty Tombs]//吴乃文 [Naiwen Wu leg.]” (CAU); one male, labeled “1956-VII-26//昌平十三陵 [China, Beijing, Changping, Ming Dynasty Tombs]//唐元麟 [Yuanlin Tang leg.]” (CAU); one male, labeled “2020.VII.29, 北京延庆刘斌堡下虎叫村 [China, Beijing, Yanqing, Liubinbu, Xiahujiao Village], Elev. 680 m, 吴超采&赠 [Chao Wu leg. and present]//河北大学博物馆 [Museum of Hebei University]”, “M33A10” (MHBUa); two females, labeled “2022.VII.12, 北京房山大石窝镇南河村 (村东部旱厕) [China, Beijing, Fangshan District, Dashiwo Town, Nanhe Village (latrine in the eastern village)]//河北大学博物馆 [Museum of Hebei University]”, “M36C8 or M36C9” (MHBUa); one male, labeled “26.VII.37//O. PIEL, coll.” (yellowish white, rectangular, handwritten and printed), “CHAHAR//Yangkiaping [China, Hebei, Zhuolu, Yangjiaping]”, “*Meloe* sp.” (NACRC); one male and one female, labeled “19.VII.37//O. PIEL, coll.”, “CHAHAR//Yangkiaping [China, Hebei, Zhuolu, Yangjiaping]”, “IOZ(E)1117513 or IOZ(E)1117514” (NACRC); one male, labeled “蜂巢 (蒙西) [beehive (China, Inner Mongolia, Mengxi)]//2001.6.25”, “IOZ(E)1117517” (NACRC); one female, labeled “蜂巢内 [in a beehive]//2001.6.25”, “IOZ(E)1117519” (NACRC); one female, labeled “蜂巢 (蒙西) [beehive (China, Inner Mongolia, Mengxi)]//2001.7.1”, “IOZ(E)1117518” (NACRC); two males, labeled “内蒙古乌海蒙西 [China, Inner Mongolia, Wuhai, Mengxi]//2001.7.1”, “IOZ(E)1117515 or IOZ(E)1117516” (NACRC); one female, labeled “宁夏盐池哈巴湖管理站 [China, Ningxia, Yanchi, Haba Lake Management Station]//37.7035N 107.0445E//1452 m, 2016-viii-3//娄康 [Kang Lou leg.]” (MHBU). All paratypes have the following label added: “PARATYPE//*Sinostenoria yangi* gen. and sp. nov.//Det. Pan Z.”.

**Etymology.** This new species is named after Prof. Jikun Yang, who previously worked at the Beijing Agricultural University (the predecessor of the China Agricultural University), to commemorate her contributions to the taxonomy of Chinese insects. Meanwhile, the holotype of this new species was collected by Prof. Yang.

**Distribution.** China (Beijing, Hebei, Inner Mongolia, Ningxia).

### 3.3. Newly Recorded Species from China

**Tribe Stenoderini Selander, 1991**

**Figure 3:**
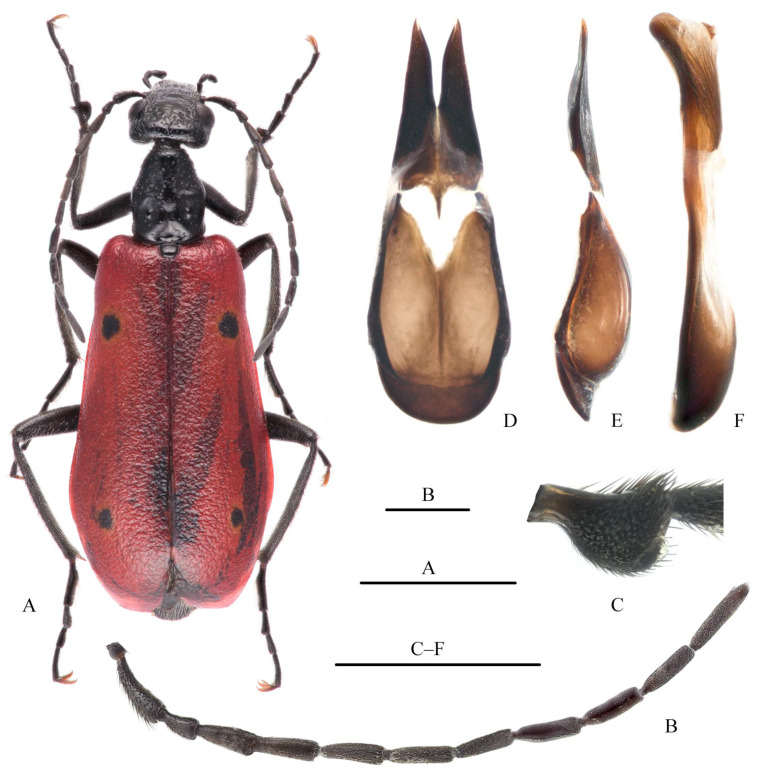
*Stenodera djakonovi*, male (from Jilin, Fengman, Zhuqueshan Park). (**A**) Habitus, dorsal view; (**B**) antenna, dorsal view; (**C**) protarsomere I, outer side, lateral view; (**D**,**E**) tegmen: (**D**) ventral view, (**E**) lateral view; (**F**) aedeagus, lateral view. Scar bars: 5 mm (**A**); 1 mm (others).

***Stenodera* (*Stenodera*) *djakonovi* Aksentjev, 1978, new record for China** (Figure 3)

*Stenodera djakonovi* Aksentjev, 1978: 124 [type locality: “Primorskij Kraj, Vinogradovka” (Primor’e Territory, Russia). Type depository: ZIN] [31].

*Stenodera* (*Stenodera*) *djakonovi*: Bologna et al. 2002: 308 [32]; Bologna, 2008: 412 [33]; 2020: 562 [34].

**Diagnosis.** This species is very close to *Stenodera foveicollis* (Fairmaire, 1897) from southeastern China. According to Bologna et al. [32], their main differences are the following: (a) head (including mouthparts) distinctly shorter than head capsule in *S. djakonovi*, rather than almost as long as head capsule of *S. foveicollis*; (b) shiny frontal area less extensive, head punctures larger, and interpunctal surfaces more opaque in *S. djakonovi*; (c) antennae of *S. djakonovi* slightly shorter than *S. foveicollis*, especially in female; (d) pronotum of *S. djakonovi* slightly wider than *S. foveicollis*; (e) punctures on pronotum of *S. djakonovi* denser than that of *S. foveicollis*. For details, see Bologna et al. [32].

**Chinese examined materials.** One female, Jilin, Jiaohe, Qingling Town, 3-V-2021, Chuhuai Zhou leg. (MHBU); one female, Jilin, Longtan District, Longtanshan Park, 2-IV-2022, Chuhuai Zhou leg. (MHBUa); one female, Jilin, Longtan District, Longtanshan Park, 8-V-2022, Chuhuai Zhou leg. (MHBU); one male one female, Jilin, Fengman, Zhuqueshan Park, elev. 390 m, 17-IV-2023, Chuhuai Zhou leg. (MHBU).

**Distribution.** China (Jilin), Russia (Primor’e Territory, Ussuri).

**Tribe Nemognathini Laporte de Castelnau, 1840**

**Figure 4:**
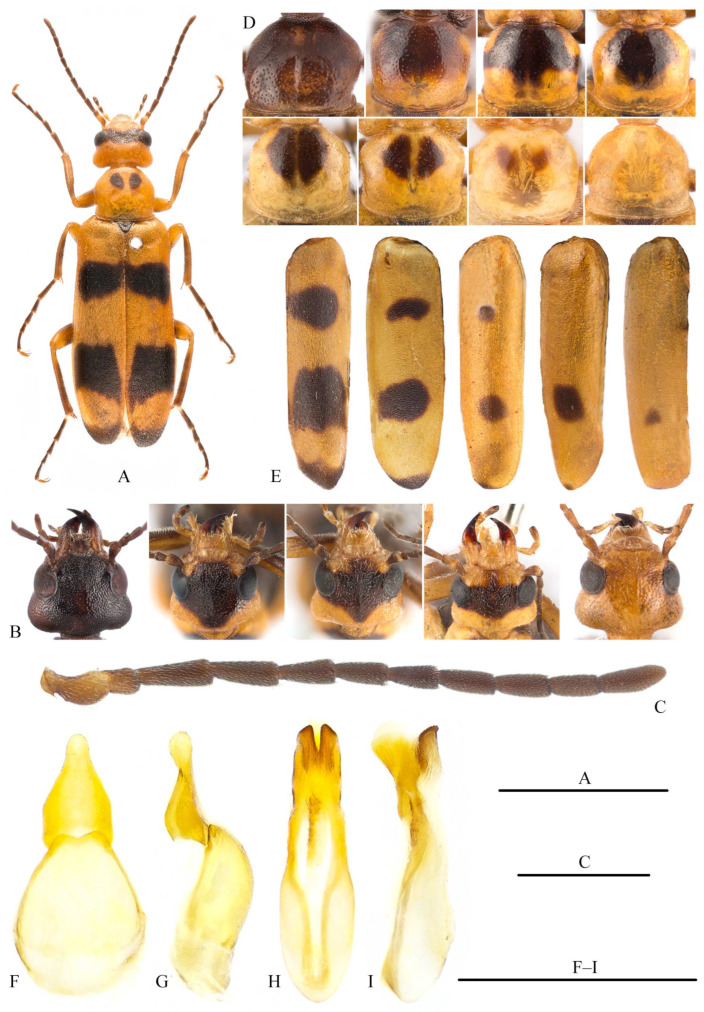
*Megatrachelus sibiricus* (from Ningxia, Mt. Yunwushan). (**A**) Habitus, male, dorsal view; (**B**) patterns of head, dorsal view; (**C**) antenna, male, dorsal view; (**D**) pronotal patterns, dorsal view; (**E**) elytral patterns, dorsal view; (**F**,**G**) tegmen: (**F**) ventral view, (**G**) lateral view; (**H**,**I**) aedeagus: (**H**) ventral view, (**I**) lateral view. Scar bars: 5 mm (**A**); 1 mm (**C**,**F**–**I**).

***Megatrachelus sibiricus* (Tauscher, 1812), new record for China** (Figure 4)

*Zonitis sibirica* Tauscher, 1812: 162 (type locality: “Russia, Sibiria”. Type depository: unknown) [35]; Gemminger and von Harold, 1870: 2161 [36]; Borchmann, 1917: 164 [37].

*Lydus quadrisignatus* Faldermann, 1835: 415 (type locality: Mongolia. Type depository: ZIN) [38]; Gemminger and von Harold, 1870: 2157 (homonym, nec Fischer von Waldheim, 1823) [36].

*Lydus quadrinotatus* Wellman, 1910: 25 (replacement name) [39]; Borchmann, 1917: 9. Synonymized by Tshernyshev and Axentiev, 1996: 55 [37].

*Megatrachelus sibirica*: Tshernyshev and Axentiev, 1996: 55 [40]; Tshernyshev, 2014: 183 (list) [41]; 2014: 415 (key) [42].

*Megatrachelus sibiricus*: Nikolaev and Kolov, 2005: 153 [43]; Bologna, 2008: 407 [33]; 2020: 555 [34].

**Redescription.** Body (Figure 4A) shiny, coloration extremely variable: head (with mouthparts) completely yellow, black, or yellow with black fasciae (Figure 4B); in completely yellow specimens, apex of mandibles reddish black and that of maxillary palpi and labial palpi dark; antennae brown-black, and most yellowish at basal one or two antennomeres (Figure 4C); pronotum completely yellow, black, or yellow with black fasciae or spots (Figure 4D); prosternum yellow to dark brown; pterothorax (including scutellum) brown-black; elytra yellow, with two black spots (only one apical spot in few cases) and a black apical fascia on each elytron (Figure 4E); legs yellow to black, but apices of tarsi always brown-black; tibial spurs reddish brown; pretarsal claws brown; abdominal ventrite I black, II to III gradually from black to yellow, IV–VI yellow. Body sparsely covered with short yellow setae. Body length: 6.5–11.5 mm; width: 2.0–3.4 mm.

Head (Figure 4B) subtriangular, slightly wider than long, with maximum width at level of temples; with a weak longitudinal ridge on vertex; densely packed with middle-sized and shallow punctures; distance between punctures less than diameter of each puncture. Temples slightly prominent; occiput protruded. Frons longitudinally convex on midline. Eyes small; minimum distance between eyes approximately 2.4× as wide as transverse width of eye, longer than temples. Clypeus short, transverse, smooth, almost impunctate. Labrum subtrapezoidal, anterior margin straight; mandibles approximately twice length of labrum and clypeus together; maxillary palpomere IV not enlarged at apex, galea short and penicillate. Antenna with 11 antennomeres, reaching posteriorly elytral base; antennomere I curved, widened at apical half; II shortest, subglobose, about half as long as III; III–XI subfiliform, at most slightly widened at apex; III longest; IV second longest; V–IX similar in length; X shorter than IX; XI subequal to IV, relatively rounded at apex.

Pronotum (Figure 4D) wider than long, widest in front of middle, narrowed anteriorly, posterior margin bordered; disc smooth, punctures smaller and sparser than those on head. Scutellum subtriangular, posterior margin rounded. Elytra distinctly bordered laterally, especially at base.

Legs not modified; pro- and mesotibial spurs spiniform; metatibial spurs with different shapes, with inner one widened and outer one more robust and wider apically; tarsomere I as long as last tarsomere, tarsomeres without pads; dorsal blade of pretarsal claws with two rows of teeth along ventral margin.

Ventrite VI deeply cleft to base in male, but shallowly V-emarginate in female. Male gonoforceps completely fused, long and campaniform in ventral view (Figure 4F), and nodular at apex in lateral view (Figure 4G). Aedeagus as in Figure 4H,I, without dorsal hooks, with two well-sclerotized ventral lobes slightly curved posteriorly; endophallus without hook.

**Diagnosis.** This species is morphologically similar to *M. politus* (Gebler, 1832), but can be easily distinguished by the elytral pattern, which includes a black apical fascia in *M. sibiricus*, lacking in the latter one. Furthermore, both the head and pronotum of *M. politus* are smoother and shinier and the punctures are finer and sparser than in *M. sibiricus*.

**Chinese examined materials.** Two females, Hebei, Zhangbei, Youlougou, 4-VIII-2000, Huaijun Xue leg. (NKU); one male, Ningxia, Mt. Luoshan, Xiongjiatang, 1816 m, 19-VII-2009, Ruilong Ma et al. leg. (MHBU); one male, Ningxia, Mt. Luoshan, western Dakouzi, elev. 1921–2187 m, 26-VIII-2009, Qi He leg. (MHBU); one male and two females, Ningxia, Guyuan, Mt. Yunwushan, Caichuan, 10-VIII-2013, Yanxia Jia and Yun Kang leg. (MHBU); six males and twenty-one females, idem., 12-VIII-2013, Xinpu Wang and Yanxia Jia leg. (MHBU); three females, Ningxia, Guyuan, Mt. Yunwushan, core zone, 9-VIII-2013, Yanxia Jia and Yun Kang leg. (MHBU); one male, idem., 12-VIII-2013, Xinpu Wang and Yun Kang leg. (MHBU).

**Distribution.** China (Hebei, Ningxia), Mongolia, Kazakhstan, Russia (Siberia).

**Taxonomic remarks.** The genus *Megatrachelus* is phylogenetically close to *Euzonitis* Semenov, 1893 (Figure 1). Until now, four species have been included in *Megatrachelus* [8,34]. Unfortunately, two of them, *M. pallidipennis* (Motschulsky, 1845) and *M. quadricollis* (Fairmaire, 1892), are poorly known to the authors. Therefore, we try to differentiate *Megatrachelus* from *Euzonitis* based on the characteristics of *M. politus* and *M. sibiricus*: (a) the outer metatibial spur is much wider than the inner spur in both genera; however, it is longer than the inner spur in *Euzonitis* and is as long as the inner one in *Megatrachelus*; (b) the lateral margin of elytra is distinctly bordered in *Megatrachelus* (the border is inflated at the base) but not bordered in *Euzonitis*; (c) the pronotum is smooth with sparse and fine punctures in *Megatrachelus* but distinctly punctate in *Euzonitis*.

**Figure 5:**
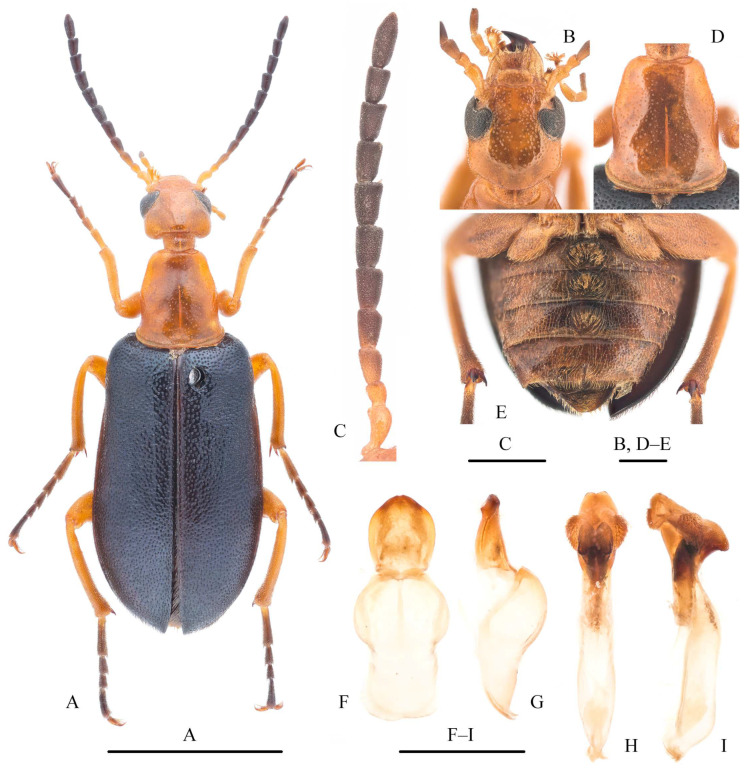
*Zonitomorpha dollei*, male (from Yunnan, Xishuangbanna, Damenglong). (**A**) Habitus, dorsal view; (**B**) head, dorsal view; (**C**) antenna, dorsal view; (**D**) pronotum, dorsal view; (**E**) abdominal ventrites, ventral view; (**F**,**G**) tegmen: (**F**) ventral view, (**G**) lateral view; (**H**,**I**) aedeagus: (**H**) ventral view, (**I**) lateral view. Scar bars: 5 mm (**A**); 1 mm (others).

***Zonitomorpha dollei* (Fairmaire, 1889), new record for China** (Figure 5)

*Zonitis dollei* Fairmaire, 1889: 366 [type locality: “Tonkin” (northern Vietnam). Type depository: MNHN] [44].

*Zonitomorpha dollei*: Pic, 1910: 391 [45]; Borchmann, 1917: 150 [37]; Pan and Ren, 2020: 265 [8].

**Redescription.** Body (Figure 5A) yellowish brown, but elytra bluish dark; apices of mandibles and tibial spurs reddish-brown; maxillary palpi and labial palpi dark brown at apex; antennae gradually darker from apex of III to XI; tarsi gradually darker from base to apex; pretarsal claws brown. Body shiny, elytra with slightly steel blue shine. Body covered by yellow setae, sparse and short in dorsal section, dense and slightly longer in ventral section; setation on elytra colored with elytra; male abdominal ventrites II–IV with a rounded depression at center, with dense and long setae inside (Figure 5E), while female without central setated depressions. Body length: 10.0–13.0 mm; width: 3.9–4.1 mm.

Head (Figure 5B) subrectangular, distinctly longer than wide, with a shallow depression on inner side of antennal socket and a longitudinal shallow furrow at center of base. Punctures middle-sized, relatively sparse; distance between punctures more than diameter of each puncture, denser towards sides. Temples slightly prominent, slightly narrower than level of eyes, lateral sides subparallel; occiput slightly protruded. Eyes small; minimum distance between eyes approximately 1.7× as wide as transverse width of eye, and slightly shorter than temples. Clypeus subtrapezoidal, wider than long, without setae or puncture at apical portion. Labrum with similar length and width to clypeus, subtrapezoidal, anterior margin straight; mandibles approximately twice as long as labrum and clypeus together; maxillary palpomeres I and II long and triangular; IV subcylindrical, galea with long and dense setae at apex. Antennae (Figure 5C) subserrate, short, posteriorly reaching elytral base; antennomere I curved, enlarged at apex; II shortest, subglobose; III–V gradually wider and longer; V–VIII similar in length; VII–VIII subequal in width and slightly wider than VI and IX; VIII–X gradually shorter; XI longest, subfusiform, approximately as wide as III.

Pronotum (Figure 5D) longer than wide (aspect ratio 1.07), campaniform; lateral sides subparallel at basal half, posterior margin boarded and widest; disc with a longitudinal furrow and a shallow depression at center, a transverse depression at fore third, and a triangular shallow depression at center of base; punctures similar to those on head but sparser. Scutellum subsemicircular, posterior margin almost straight. Legs not modified; tibial spurs dorsally slightly curved at apex; pro- and mesotibial spurs spined, metatibial spurs widened; protibial inner spur longer and wider than outer one; meso- and metatibial outer spurs longer and wider than inner spurs; pro- and mesotarsomere V longest and I second longest, metatarsomere I longest and IV second longest; dorsal blade of pretarsal claws with two rows of teeth along ventral margin.

Ventrite V almost straight on posterior margin; ventrite VI divided into two lobes in male, while in female, not divided and only concave in middle of posterior margin. Male gonoforceps almost completely fused, only slightly concave in middle of apex (Figure 5F); each side of gonoforceps with a tooth on dorsal margin (Figure 5G); gonocoxal plate approximately twice length of gonoforceps (Figure 5F,G). Aedeagus as in Figure 4I, without dorsal hooks, but with two ventral lobes curved posteriorly; endophallus without hook.

**Diagnosis.** *Zonitomorpha dollei* is very close to *Z. melanoptera* Fairmaire, 1894, from Bangladesh, but the latter has a relatively wider pronotum, elytra not distinctly widened at apex, and antennae relatively slender and light colored.

**Type specimens examined.** One ex., with the following labels: “*Zonitis Dollei* Fairmaire//Tonkin”, “*Zonitomorpha dollei* (Fairm)//M. A. Bologna det. 2016” (MNHN; see Figure 5 in Pan and Ren, 2020 [8]).

**Chinese materials examined.** Two females, Yunnan, Xishuangbanna, Damenglong, elev. 650 m, 4-VIII-1958, Leyi Zheng, Yiran Zhang leg. (NACRC); one male, one female, Yunnan, Xishuangbanna, Damenglong, elev. 650 m, 8-VIII-1958, Yiran Zhang leg. (NACRC); one male, Yunnan, Xishuangbanna, Yunjinghong, elev. 650 m, 27-VIII-1958, Yiran Zhang leg. (NACRC).

**Distribution.** China (Yunnan), Vietnam.

### 3.4. Review of the Genus Oreomeloe Tan, 1981

**Genus *Oreomeloe* Tan, 1981**

*Oreomeloe* Tan, 1981: 411. Type species: *Oreomeloe spinulus* Tan, 1981, by original designation, by monotypy [46].

**Distribution.** China (Xizang).

**Figure 6:**
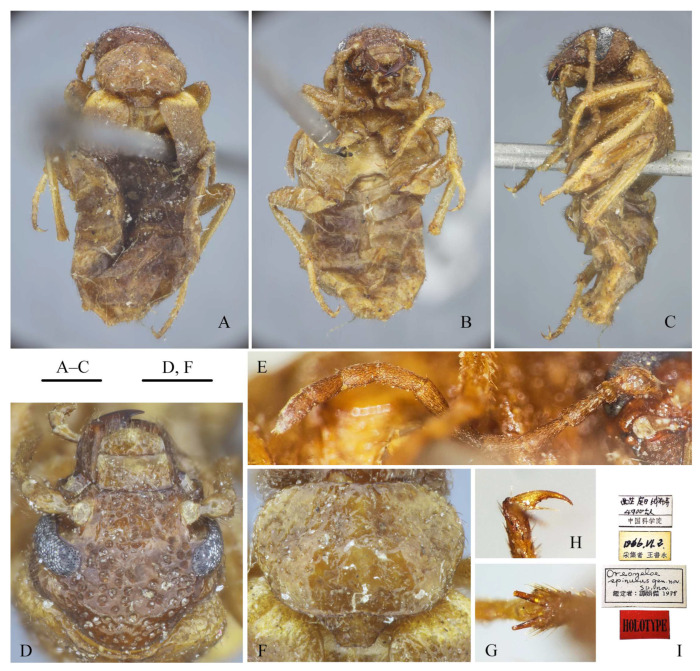
*Oreomeloe spinulus*, holotype, female. (**A**–**C**) Habitus: (**A**) dorsal view, (**B**) ventral view, (**C**) lateral view; (**D**) head, dorsal view; (**E**) antenna; (**F**) pronotum and scutellum, dorsal view; (**G**) metatibial spurs, left; (**H**) metapretarsal claws, left; (**I**) specimen labels (translations see the Type specimens examined). Scar bars: 1 mm (**A**–**C**); 0.5 mm (**D**,**F**).

***Oreomeloe spinulus* Tan, 1981** (Figure 6)

*Oreomeloe spinulus* Tan, 1981: 415 (type locality: Rongbuk Monastery, Tingri, Xizang, China. Type depository: NACRC) [46]; Bologna, 2008: 404 [33]; 2020: 550 [34]; Pan and Ren, 2018: 79 [47].

**Type specimens examined.** Holotype: female, labeled “西藏定日绒布寺 [China, Xizang, Tingri, Rongbuk Monastery]//4900 公尺 [4900 m]//中国科学院 [Chinese Academy of Sciences]”, “1966.VI.2//采集者 王书永 [Shuyong Wang leg.]”, “*Oreomeloe spinulus* gen. nov., sp. nov.//鉴定者: 谭娟杰 1978 [Det. Juanjie Tan, 1978]”, “HOLOTYPE” (NACRC). Paratypes: one female, labeled “西藏定日绒布寺 [China, Xizang, Tingri, Rongbuk Monastery]//4900 m, 1 female//中国科学院 [Chinese Academy of Sciences]”, “1966.VI.2//采集者 王书永 [Shuyong Wang leg.]”, “PARATYPE” (NACRC); one female, labeled “西藏聂拉木亚里 [China, Xizang, Nyalam, Yali]//4400 米 [4400 m]//中国科学院 [Chinese Academy of Sciences], 1 female”, “1966.VI.18//采集者 王书永 [Shuyong Wang leg.]”, “PARATYPE” (NACRC).

**Distribution.** China (Xizang).

**Remarks.** Tan [46] described a high mountain-adapted genus and species, *Oreomeloe spinulus*, from Xizang and considered it to be close to *Meloe* Linnaeus, 1758. This genus was formally positioned in the tribe Meloini Gyllenhal, 1810, by Selander [48], and this inclusion was accepted by other studies [5,8,33,34,47,49]. However, nobody could understand what this monotypic genus is. In April 2023, one of the authors (Z. P.) found all type specimens of *O. spinulus* (Figure 6) at NACRC. After the examination, we are now proposing that this genus must be positioned in the tribe Nemognahini rather than in Meloini for the following combined reasons: (a) the body (Figure 6A–C) is brown-yellow (most species of Meloini have a black or blue body, except the Moroccan species *Meloe pallidicolor* Martinez de la Escalera, 1909); (b) the pretarsal claws (Figure 6H) are extremely reduced, almost setiform, and the greatest width of its ventral blade is distinctly less than the half width of the dorsal blade (the ventral blade is wider than the half width of the dorsal blade in Meloini); (c) the elytra (Figure 6A) are widely separated immediately at the base (the elytra are not distinctly separated and sometimes overlapping at the base in Meloini).

Interestingly, Schawaller [50] discovered one sexually dimorphic species, *Stenoria thakkhola* Schawaller, 1996, from Mustang District in Nepal, a locality not far from the type locality of *O. spinulus*. The female of *S. thakkhola* has relatively short antennae, reduced elytra, and absent hind wings similarly to in *O. spinulus*. The female of these two species could be distinguished only by their body coloration and antennal length: (a) the head, antennae, thorax, coxae, and femora are yellow to brown in *O. spinulus* (the integument is well sclerotized) but black in *S. thakkhola*; (b) the elytra of *O. spinulus* are brown but yellow at the base, while those of *S. thakkhola* are black on the outer half and yellow-orange on the inner half; (c) the antennae of *O. spinulus* are relatively elongate, posteriorly reaching the metacoxae, and antennomeres V–XI are slightly shorter than IV, but the antennae of *S. thakkhola* are short, only posteriorly reaching the base of the pronotum. Antennomeres V–XI are shortened, distinctly shorter than IV. For these reasons, we suspect that *O. spinulus* could be close to *S. thakkhola*. This does not rule out the possibility that they belong to the same genus, because male of *S. thakkhola* have typical, slightly reduced elytra as in congeneric species. This problem will be resolved by the discovery of males of *O. spinulus*. Moreover, as previously discussed, the revision of the genus *Stenoria*, polyphyletic in the present status [2], is necessary to understand the true relationships of several species from Eurasia and Africa, now referred to in this genus, which shows a great variation is some morphological features.

Additionally, the data of one of the paratypes were erroneously recorded by Tan [46]. She recorded both paratypes as collected in Nyalam, while one of them is from the type locality.

## 4. Discussion

The evolutionary history of Nemognathinae was delineated by Riccieri et al. [2] based on five molecular markers, which covered all tribes and most genera. Although that study was extensive, 11 genera remain to be analyzed, including *Longizonitis*. Pan et al. [6] failed to outline the definitive placement of this genus and instead observed a patchwork of similarities, including (a) with the *Nemognatha* lineage (which includes *Palaestrida* White, 1846, and some Nearctic *Nemognatha* Illiger, 1807, subgenera), according to its short antennomere II, a typical condition of the tribe Palaestrini and of a few Nemognathini (see also the present study); (b) with New World *Zonitis*, *Pseudozonitis* Dillon, 1952, and *Gnathium* Kirby, 1818, as well as some Palaearctic *Zonitis* species, because of the ventral sclerotized lobes of the aedeagus; (c) with the sitarine lineage due to the lack of great modification of its galeae. In contrast to the above hypotheses, the present molecular phylogenetic result shows that *Longizonitis* is grouped with a portion of the polyphyletic *Stenoria* and is quite closely related to *Ctenopus* Fischer von Waldheim, 1823.

Interestingly, the new genus, *Sinostenoria*, is classified in the same clade as *Longizonitis*, but this clade is not supported by synapomorphies. *Sinostenoria* and *Allendesalazaria* Martinez de la Escalera, 1910 share comparable morphological characteristics. However, in *Allendesalazaria*, the ventral blade of the claws is absent, and the elytra are strongly dehiscent at the base immediately beyond the scutellum. The females of *Sitarobrachys* Reitter, 1883 and *Oreomeloe*, as well as *Stenoria thakkhola*, have reduced elytra as well, but their males have partially reduced elytra and wings, except for the unknown male of *Oreomeloe*. *Sitarobrachys* can be easily distinguished from others by the presence of teeth along the ventral margin of the dorsal blade of the claws. The female of *Sinostenoria* can be distinguished from the females of *Oreomeloe* and *Stenoria thakkhola* by its antennae with 10 antennomeres and elytra that are not remarkably divided at the base. Furthermore, *Sitaromorpha* Dokhtouroff, 1890 is a mysterious taxon because it was only described in a single female (the original description is wide and detailed but does not include enough key characteristics to define this genus) [51] and has not been collected since the original work. According to the description in [51], *Sitaromorpha* seems to be very close to *Sinostenoria*, but its last three antennomeres are fused and its elytra immediately separate at the base.

The dorsal blade of the claws having one or two rows of teeth along the ventral margin is a typical feature of Nemognathini. However, in certain “sitarine” taxa, such as *Oreomeloe*, *Sinostenoria*, *Sitaromorpha*, and a few species of *Sitaris* Latreille, 1810, the dorsal blade of the claws is smooth along ventral margin. Bologna and Pinto [5] mistakenly placed *Sitaromorpha* in the group with teeth along the ventral margin of the dorsal blade. Additionally, other taxa have only partially reduced elytra, especially on the sides, such as *Nyadatus* Aksentjev, 1981, *Stenoria laterimaculata* (Reitter, 1898), and *Stenoria analis* Schaum, 1859, and some *Sitaris* also have such a derived condition. This pattern suggests similarities among these taxa. Unfortunately, none of them have been included in either previous or present phylogenetic analyses, except *Sinostenoria* and *Stenoria analis*. We anticipate that the relationships will be clarified by the discovery of more material, e.g., the larvae, the males of *Oreomeloe* and *Sitaromorpha*, and molecular data.

## 5. Conclusions

In this study, a new genus and a new species from northern China were described and illustrated as *Sinostenoria yangi* Pan, **n. gen. and sp.** Three newly recorded species, *Megatrachelus sibiricus* (Tauscher, 1812), *Zonitomorpha dollei* (Fairmaire, 1889), and *Stenodera djakonovi* Aksentjev, 1978, were also discussed and illustrated, with specimens collected from various regions in China.

In addition, we proposed that the genus *Oreomeloe* needs to be transferred from the tribe Meloini to the Nemognathini after examining the types. A molecular phylogenetic analysis supported the systematic placement of *Sinostenoria* and *Longizonitis* in a clade with *Stenoria* cf. *grandiceps* and *Ctenopus* cf. *persicus*. However, the species diversity of Chinese Nemognathinae and the phylogenetic relationships among nemognathine taxa still require further investigation and evidence for a better resolution.

We suggest that further research and the gathering of additional evidence will contribute to resolving these gaps and lead to a more thorough understanding of the species diversity and phylogenetic relationships within the Chinese Nemognathinae.

## Data Availability

The molecular data produced in this work are available in GenBank under the following accession numbers: *COI* PP415775–PP415776; *16SrRNA* PP414784–PP414785; *28SrRNA* PP417813–PP417814; *CAD* PP415777; *ITS2* PP440018–PP440019.

## Supplementary Materials

The following supporting information can be downloaded at: https://www.mdpi.com/article/10.3390/insects15050338/s1, Table S1: Species, specimen codes, sample localities, genes, and GenBank accession numbers for the specimens included in this study.

## Appendix A

**A species list of the subfamily Nemognathinae from China.**

**Tribe Stenoderini Selander, 1991**

**Genus *Stenodera* Eschscholtz, 1818**

*Stenodera* (*Stenodera*) *djakonovi* Aksentjev, 1978, **new record for China**—China (Jilin); Russia.

*Stenodera* (*Stenodera*) *foveicollis* (Fairmaire, 1897)—China (Jiangxi, Fujian).

**Tribe Horiini Latreille, 1802**

**Genus *Horia* Fabricius, 1787**

*Horia debyi* (Fairmaire, 1885)—China (Yunnan); India, Sri Lanka, Philippines, Malaysia, Indonesia.

**Genus *Synhoria* Kolbe, 1897**

*Synhoria maxillosa* (Fabricius, 1801)—China (Henan, Taiwan, Hongkong, Sichuan); Japan, India, Indonesia, Thailand, Papua New Guinea, NE Australia.

**Tribe Nemognathini Latreille, 1840**

**Genus *Apalus* Fabricius, 1775**

*Apalus davidis* (Fairmaire, 1886)—China (Zhejiang, Hubei).

*Apalus haemapterus* Fairmaire, 1889—China (Shandong, Sichuan).

Genus *Ctenopus* Fischer von Waldheim, 1823

*Ctenopus eous* Semenov, 1900—China (Xinjiang).

*Ctenopus lama* Escherich, 1904—China (Xinjiang).

*Ctenopus semenovi* Reitter, 1895—China (Xinjiang).

*Ctenopus sinuatipennis* (Fairmaire, 1892)—China (Xinjiang); Turkmenistan, Uzbekistan.

**Genus *Euzonitis* Semenov, 1893**

*Euzonitis quadrimaculata* (Pallas, 1782)—China (Inner Mongolia, Ningxia, Xinjiang); Mongolia, Central Asia west to Portugal, North Africa.

**Genus *Glasunovia* Semenov, 1896**

*Glasunovia sinica* Pic, 1938—China.

**Genus *Longizonitis* Pan** **and Bologna, 2018**

*Longizonitis semirubra* (Pic, 1911)—China (Fujian, Yunnan, Xizang); India.

**Genus *Megatrachelus* Motschulsky, 1845**

*Megatrachelus politus* (Gebler, 1832)—China (Hebei, Inner Mongolia, Shanghai, Northeast Territory); Japan, Korea, Mongolia, Russia.

*Megatrachelus sibiricus* (Tauscher, 1812), **new record for China**—China (Hebei, Ningxia), Mongolia, Kazakhstan, Russia.

**Genus *Oreomeloe* Tan, 1981, new transfer from Meloini**

*Oreomeloe spinulus* Tan, 1981—China (Xizang).

**Genus *Sinostenoria* Pan, gen. nov.**

*Sinostenoria yangi* Pan, **sp. nov.**—China (Beijing, Hebei, Inner Mongolia, Ningxia).

**Genus *Sitaris* Latreille, 1802**

*Sitaris* (*Sitaris*) *pectoralis* Bates, 1879—China (Xinjing).

**Genus *Stenoria* Muslsant, 1857**

*Stenoria* (*Stenoria*) *fasciata* (Faldermann, 1835)—China (Heilongjiang, Inner Mongolia, Hebei); Mongolia.

*Stenoria* (*Stenoria*) *hauseri* (Escherich, 1904)—China (Qinghai, Xinjiang, Xizang).

*Stenoria* (*Stenoria*) *laterimaculata* Reitter, 1898—China (Inner Mongolia); Mongolia, Tajikistan.

*Stenoria* (*Stenoria*) *longipennis* (Pic, 1933)—China (Gansu).

*Stenoria* (*Stenoria*) *tibetana* (Escherich, 1904)—China (Qinghai, Xizang).

**Genus *Zonitis* Fabricius, 1775**

*Zonitis* (*Zonitis*) *ballionis* Escherich, 1892—China (Xinjiang); Afghanistan, Kazakhstan, Kyrgyzstan, Tajikistan, Turkmenistan, Uzbekistan.

*Zonitis* (*Zonitis*) *flava* Fabricius, 1775—China (Xinjiang); Central Asia west to Portugal, North Africa.

*Zonitis* (*Zonitis*) *fortuccii* Fairmatre, 1887—China (Beijing, Hebei); Mongolia.

*Zonitis* (*Zonitis*) *koslowi* Semenov, 1900—China (Xinjiang).

*Zonitis* (*Zonitis*) *sinensis* Pic, 1935—China (Fujian, Sichuan).

**Genus *Zonitomorpha* Péringuey, 1909**

*Zonitomorpha davidis* (Fairmaire, 1886)—China (Beijing, Shandong, Taiwan).

*Zonitomorpha miwai* (Kôno, 1936)—China (Taiwan).

*Zonitomorpha dollei* (Fairmaire, 1889), **new record for China**—China (Yunnan); Vietnam.

**Genus *Zonitoschema* Péringuey, 1909**

*Zonitoschema angustithorax* (Pic, 1912)—China (Shandong).

*Zonitoschema cothurnatum* (Marseul, 1873)—China (Zhejiang, Taiwan); Korean Peninsula, Japan.

*Zonitoschema elongatipenne* (Pic, 1915)—China (Jiangxi).

*Zonitoschema fuscimembre* (Fairmaire, 1886)—China (Fujian, Yunnan).

*Zonitoschema japonicum* (Pic, 1910)—China (Gansu, Shanghai, Zhejiang, Taiwan); Korean Peninsula, Japan.

*Zonitoschema klapperichi* Borchmann, 1941—China (Fujian).

*Zonitoschema macroxanthum* (Fairmaire, 1887)—China (Shaanxi, Zhejiang); Philippines, Indonesia.

*Zonitoschema stramineum* (Fairmaire, 1894)—China (Fujian); India.

*Zonitoschema yunnanum* Kaszab, 1960—China (Yunnan).
