# Supplementary material for: Contributions to the Knowledge of Nemognathinae (Coleoptera: Meloidae) from China
Source: Insects. 2024 May 8;15(5):338. doi: 10.3390/insects15050338 (PMC11122394; doi:10.3390/insects15050338)
Supplement: Supplementary file 1 [file insects-15-00338-s001.zip › insects-2978189-supplementary.pdf]

Table S1. Species, Specimen code, sample localities, genes, and GenBank accession numbers for the specimens included in this study.

**Species**	**Specimen Code**	**Collection Data**	***COI***	***16S rRNA***	***28S rRNA***	***CAD***	***ITS2***
Longizonitis semirubra	M7G4	China, Xizang, Yadong, Xiayadong, 2017.VI.23, Jian-Yue Qiu and Hao Xu leg.	PP415776	PP414785	PP417814	-	PP440019
Sinostenoria yangi	M36C9	China, Beijing, Fangshan District, Dashiwo Town, Nanhe Village (latrine in the eastern village), 2022.VII.12	PP415775	PP414784	PP417813	PP415777	PP440018
